# Mechanisms driving diversity–productivity relationships differ between exotic and native communities and are affected by gastropod herbivory

**DOI:** 10.1007/s00442-015-3395-2

**Published:** 2015-08-04

**Authors:** Lotte Korell, Robin Schmidt, Helge Bruelheide, Isabell Hensen, Harald Auge

**Affiliations:** Institute of Biology, Am Kirchtor 1, 06108 Halle, Germany; Department of Community Ecology, Helmholtz Centre for Environmental Research - UFZ, Theodor-Lieser-Straße 4, 06120 Halle, Germany; Institute of Biology, Philipps-University Marburg, Karl-von-Frisch-Straße 8, 35043 Marburg, Germany; German Centre for Integrative Biodiversity Research (iDiv) Halle-Jena-Leipzig, Deutscher Platz 5e, 04103 Leipzig, Germany

**Keywords:** *Arion vulgaris*, Complementarity effect, Evenness, Functional groups, Selection effect

## Abstract

Biodiversity experiments have shown that productivity usually increases with plant species richness. However, most of those studies disregarded the importance of trophic interactions to the diversity–productivity relationship, and focused on the loss of native species while ignoring invasions by exotic species. Yet, as functional complementarity and the impact of plant antagonists are likely to differ between native and exotic communities, the diversity–productivity relationship may change when native communities are invaded by exotic species. We conducted a mesocosm experiment to test how diversity effects, evenness, and productivity differed between exotic and native assemblages of grassland plants, and how these communities were influenced by slug herbivory. In line with other experiments, we found higher productivity in exotic than in native communities. However, different mechanisms (complementarity vs. selection effect) contributed to the positive diversity–productivity relationships in exotic vs. native communities. Against expectations, native communities showed much lower evenness and a greater selection effect, suggesting that competitive dominance among native species may be even stronger than among exotic species. Slug herbivory decreased productivity independently of species origin and species diversity. However, exotic communities showed a threefold higher complementarity effect than native communities in the absence of slugs, which was mainly driven by differences in the responses of native and exotic legumes and nonleguminous herbs. Our results imply that underlying mechanisms for the positive diversity–productivity relationship differ between native and exotic communities in the early stages of community development, and that differential responses of plant functional groups to generalist herbivory can contribute to this pattern.

**Electronic supplementary material:**

The online version of this article (doi:10.1007/s00442-015-3395-2) contains supplementary material, which is available to authorized users.

## Introduction

The invasive spread of exotic species can lead to the homogenization of local communities, which in turn can have far-reaching consequences for ecosystem functioning (Olden et al. 2004). However, many exotic plant species integrate into resident communities without much ecological impact and do not reduce—or may even increase—local diversity (e.g., Sax and Gaines 2003; Ortega and Pearson 2005; Maron et al. 2014). Other exotics may become dominant and outcompete natives, thereby reducing diversity but often increasing productivity compared to noninvaded communities (Hejda et al. 2009; Vilà et al. 2011). Such comparisons have, however, mostly been correlative (but see Wilsey et al. 2009; Maron et al. 2014), and may thus be confounded with covarying environmental factors.

Experiments investigating the relationship between plant diversity and various ecosystem processes such as primary production have mostly been confined to native species, or have arbitrarily included some common exotic species without taking species origin explicitly into account (e.g., Dukes 2001; Reich et al. 2001; Fridley 2002). These biodiversity experiments have consistently shown that plant species richness has a positive effect on productivity (Hooper et al. 2005; Cardinale et al. 2011). This positive effect of plant diversity on productivity was also shown for “real” grassland ecosystems (Stein et al. 2008), and is usually explained by two not mutually exclusive processes: the complementarity effect (CE) and the selection effect (SE) (Loreau and Hector 2001). A positive CE indicates that species have higher productivity on average in a mixture than expected from monoculture, but it does not measure resource partitioning between them (Carroll et al. 2011; Loreau et al. 2012). In contrast, SE indicates that species with certain traits, e.g., a particularly low or high monoculture biomass, are favored in species mixtures (Hooper et al. 2005). However, diversity–productivity relationships have rarely been compared between native and invaded or exotic-dominated communities (Maron and Marler 2008; Wilsey et al. 2009). Recently, it has been demonstrated that mechanisms facilitating biodiversity maintenance and stability differ between native and exotic-dominated communities, but the results are not unequivocal. While Isbell and Wilsey (2011) found that increasing species richness from one to four species enhanced aboveground productivity in native but not in exotic grassland communities, Wilsey et al. (2009) and Cook-Patton and Agrawal (2014) revealed a positive effect of diversity on productivity for both native and exotic communities. Moreover, communities composed of exotic grassland species showed a strong positive SE and low evenness while native communities showed a strong positive CE and higher evenness in some studies (Wilsey et al. 2009, 2014), whereas both exotic and native mixtures displayed strong CE in another study (Cook-Patton and Agrawal 2014). These patterns suggest that diversity–productivity relationships and the mechanisms driving diversity–productivity relationships may differ between native and exotic communities.

Plant antagonists such as herbivores or pathogens may modify (Mulder et al. 1999; Thébault and Loreau 2005; Duffy et al. 2007) or even drive the positive relationship between diversity and productivity (Maron et al. 2011; Schnitzer et al. 2011; Cook-Patton et al. 2014). Negative biotic feedback mechanisms might result in steeper slopes of the biodiversity–productivity functioning relationship because species-specific negative interactions increase disproportionally with increasing density of target plants (Schnitzer et al. 2011). However, previous biodiversity experiments have neglected the role of multiple trophic levels, which are common in real ecosystems (but see Scherber et al. 2006). The impact of herbivores on species richness and evenness is frequently positive (Hillebrand et al. 2007) but also depends on the type of herbivore and the diversity and productivity of the plant community (Olff and Ritchie 1998; Koricheva et al. 2006; Schuldt et al. 2010). The influence of invertebrates has been underestimated for a long time (Allan and Crawley 2011), but an increasing number of studies are showing that invertebrates can have as important effects as vertebrate herbivores on plant community structure and composition (e.g., Hulme 1994; Bruelheide and Scheidel 1999). Herbivory by insects (which are often specialists) was often observed to promote diversity and evenness in herbaceous plant communities by suppressing dominant species and allowing competitively inferior species to coexist (Carson and Root 2000; Stein et al. 2010; Allan and Crawley 2011). In contrast, herbivory by invertebrate generalists, such as gastropods, can lead to a change in plant community composition towards dominant species at the expense of subdominant species (Hulme 1994; Hanley 2004), which in turn may result in reduced species richness (Allan and Crawley 2011, but see Buschmann et al. 2005; Peters 2007; Motheral and Orrock 2010).

If exotic plant species are dislocated from coevolved relationships with specialized natural enemies (Hallett 2006), we may expect fundamental differences in the role that specialists play in the diversity–productivity relationship between native and exotic plant communities. In contrast, differences in how generalist enemies may affect exotic compared to native plant communities remain less obvious and may depend on the introduction history of the exotic plants. It is often assumed that a lack of specialists and the presence of generalists in a particular plant community selects for increased resistance to generalists (Joshi and Vrieling 2005; Liao et al. 2014), but if exotics are introduced as forage crops, generalist herbivores may have a stronger impact on exotics than on natives (Isbell and Wilsey 2011). When considering the effects of generalists such as gastropods on plant community attributes, however, we may expect similar effects on both native and exotic-dominated communities, as generalists usually show grazing preferences that depend on the traits of the plant species involved (Olff and Ritchie 1998; Scheidel and Bruelheide 1999; Allan and Crawley 2011).

In spite of the impacts that invasions by exotic species may have on ecosystem functions and services, there are surprisingly few studies that have compared the functional role of generalist herbivores in exotic-dominated and native plant communities. We therefore conducted a mesocosm experiment to investigate the effects of gastropod herbivory on monocultures and mixtures of grassland plant species that are either native or exotic to Central Europe. In particular, we were interested in two questions. How do native and exotic communities differ in evenness and the relationship between species richness and productivity? How do generalist herbivores affect the relationship between diversity and productivity in native as compared to exotic plant communities? We expected native species mixtures to show a stronger CE and higher evenness, and exotic mixtures to show a lower evenness and stronger SE, because some of the exotics should be able to gain competitive dominance. Furthermore, we expected gastropods to reduce evenness by selectively feeding on subdominant plant species, irrespective of the native or exotic origins of plant species mixtures.

## Materials and methods

### Experimental design

Our experiment was carried out in an unheated greenhouse at the experimental station of the Helmholtz Centre for Environmental Research in Bad Lauchstädt, Central Germany (51°23′38″N, 11°52′45″E). We used a randomized block design with four factors: plant species origin (exotic vs. native), diversity, species composition nested within diversity, and herbivory. The species pool of our experiment comprised 12 native and 12 exotic plant species (Table 1, Online resource 1 in the Electronic supplementary material, ESM), all of them occurring in mesic or semi-dry grasslands or in ruderal vegetation of Central German lowlands. Species were selected to represent phylogenetic pairs of one native and one exotic species within three functional groups (grasses, legumes, and nonleguminous herbs; Online resource 1 in the ESM). This pairing was done to prevent the confounding effects of functional group and phylogenetic affiliation (Agrawal and Kotanen 2003; Wilsey et al. 2009). Our exotic species pool included invasive as well as noninvasive species native to various geographic regions. Seeds of all species were collected in wild-growing populations in Central Germany, except for seeds of *Lupinus polyphyllus*, which originated from cultivated populations. We considered two levels of diversity: monocultures of all 24 species and mixtures of either six native or six exotic species. Using the random partitions approach (Bell et al. 2009), which ensures the same frequency of all species across mixtures, we randomly selected six native and six exotic species combinations. Each species combination was constrained to consist of one grass species, two legume species, and three nonleguminous herb species. By selecting native–exotic species pairs together, we obtained six phylogenetically adjusted pairs of native and exotic species mixtures. Thus, there were 12 different phylogenetically adjusted compositions of grass, herb, and nonleguminous herb species in total. To test for the effects of generalist herbivory, each monoculture and each species mixture was subjected to a control treatment without herbivory and an herbivory treatment, using the widespread slug species *Arion vulgaris* as a generalist herbivore. This slug is known to cause severe damage to wild and cultivated plants (Keller et al. 1999; Buschmann et al. 2005; Pfenninger et al. 2014). In our area, it is among the most abundant herbivorous slug species (Korell et al., pers. observation). We set up two replicates of each treatment combination, resulting in a total of 144 mesocosms [2 origins × (12 monocultures + 6 mixtures) × 2 herbivory treatments ×  2 replicates].

**Table 1:** Species pool used for the experiment

Functional group	Exotic species	Life span^d^	Origin of exotic species		Native species	Life span^d^	Shared taxon
Grasses	*Bromus tectorum*	A	Europe, western Asia^d^		*Bromus hordeaceus*	A	Genus *Bromus*
	*Lolium multiflorum*	A, B, mP	(Sub-)mediterranean Europe, western Asia^b, c, d^		*Dactylis glomerata*	pP	Subfamily Pooideae
Leguminous herbs	*Medicago* x *varia* ^a^	pP	Hybrid between native *M. falcata* and west-Asian *M. sativa* ^b, c, d^		*Medicago falcata*	pP	Genus *Medicago*
	*Onobrychis viciifolia* ^a^	pP	Mediterranean Europe, western Asia^b, c, d, e^		*Onobrychis arenaria*	pP	Genus *Onobrychis*
	*Vicia villosa* ^a^	A	Mediterranean Europe, western Asia^c, d^		*Vicia cracca*	pP	Genus *Vicia*
	*Lupinus polyphyllus* ^a^	pP	Western North America^b, c, d^		*Lotus corniculatus*	pP	Subfamily Faboideae
Nonleguminous herbs	*Foeniculum vulgare*	B, pP	Mediterranean Europe, western Asia^c, d^		*Falcaria vulgaris*	pP	Subfamily Apioideae
	*Pimpinella peregrina*	pP	(Sub-)Mediterranean Europe, western Asia^c, d^		*Pimpinella saxifraga*	pP	Genus *Pimpinella*
	*Senecio inaequidens*	pP	Southern Africa^c, d, e^		*Inula salicina*	pP	Subfamily Asteroideae
	*Solidago canadensis*	pP	North America^c, d^		*Tragopogon dubius*	B	Family Asteraceae
	*Sanguisorba minor* ssp*. polygama*	pP	Southern Europe, western Asia^c^		*Sanguisorba minor ssp. minor*	pP	Species *Sanguisorba minor*
	*Dianthus giganteus*	pP	South-eastern Europe, western Asia^c, e^		*Dianthus carthusianorum*	pP	Genus *Dianthus*

Each exotic species is shown with its paired native species. The shared taxon is the lowest common taxonomic unit of the respective species pair

*A* annual, *B* biennial, *mP* monocarpic perennial, *pP* polycarpic perennial

^a^Indicates escaped and naturalized fodder or forage species (Hanelt 2001)

Sources: ^b^Bundesamt für Naturschutz: FloraWeb—Daten und Informationen zu Wildpflanzen und zur Vegetation Deutschlands. URL: http://www.floraweb.de (11 February 2015)

^c^Das nationale Daten- und Informationszentrum der Schweizer Flora: Info Flora. URL: https://www.infoflora.ch/ (11 February 2015)

^d^Kühn et al. (2004)

^e^USDA, ARS, National Genetic Resources Program: Germplasm Resources Information Network (GRIN). National Germplasm Resources Laboratory, Beltsville, MD, USA. URL: http://www.ars-grin.gov (11 February 2015)

In late March 2012, seedlings of the 24 plant species were raised in QuickPot^®^ containers filled with a 3:1 mix of potting soil and sand. If appropriate, propagules were exposed to a 6-week stratification treatment at 5 °C or scarified with fine sandpaper prior to sowing. In early May, approximately 5 weeks after germination, seedlings were transplanted into mesocosms filled with soil from a nearby field site (0–30, chernozem, Altermann et al. 2005). Twelve seedlings were planted in each container (22 cm diameter, 25 cm depth, 7 L volume) using a regular spatial pattern. In mixtures, 2 individuals of each contributing species were randomly assigned to the 12 planting positions. Mesocosms were transferred to an unheated greenhouse equipped with mobile roofs and windows to ensure optimal ventilation and illumination, and placed on six transportation carts with special turnstiles which allowed the positions of the plant containers to be changed. Carts were grouped into two blocks, each block containing one replicate of each treatment combination. Mesocosms were randomly assigned to the 24 positions on each cart, with the restriction that herbivory and control treatments of a given monoculture or mixture were placed on the same cart. Until 2 weeks after planting, dead individuals were replaced. All mesocosms were watered manually on a regular basis to maintain approximately 60 % of field capacity. To assure similar conditions for all mesocosms, their positions were randomly changed once a week by turning the turnstiles on the carts.

The herbivory treatment started 4 weeks after planting. Slugs were trapped at a grassland site close to the greenhouse and had a size of 3–5 cm stretched. We placed one slug in each mesocosm scheduled for the herbivory treatment. In order to prevent slugs from escaping, each mesocosm was caged with sleeves of curtain fabric mounted on 1 m tall metal frames. To assure similar microclimate and light conditions for all mesocosms, those without herbivory were equipped with the same cages. In addition to regular watering, mesocosms were sprayed with water every day to improve the moisture supply for slugs. For the whole duration of the herbivory treatment, we checked all mesocosms for the presence of slugs several times each week. Escaped or dead slug individuals were replaced immediately to ensure constant herbivore pressure. The experiment was terminated in late July 2012, approximately 10 weeks after establishing the mesocosms and 6 weeks after starting the herbivory treatment. At this point in time, the annuals in our species pool (Table 1) reached maximum biomass, produced seeds, and were about to senesce. In spite of the relatively short duration of the experiment, the plants were apparently large enough to interact both above- and belowground. Furthermore, the timing of our experiment mimicked the temporal sequence of growth and management of Central European grasslands (which are typically managed by mowing or by grazing): the growing period of grasslands starts in April, and the first mowing usually takes place in May or June. We harvested the aboveground biomass of all mesocosms and separated it by species. Belowground biomass was cleaned with tap water but could not be separated by species. The aboveground biomass and belowground biomass were dried for 48 h at 60 °C and weighed. The total biomass of each mesocosm was calculated by adding the aboveground and belowground biomass.

### Statistical analysis

Based on the aboveground biomass, we used various measures to quantify the effect of diversity on the productivity of our mesocosms. First, we partitioned the net diversity effect into its additive components, the complementarity effect (CE) and the selection effect (SE) (Loreau and Hector 2001). The CE compares the growth of species in a mixture to their growth in monoculture, and is related to functional complementarity among species. The SE indicates that species with either a particularly high monoculture biomass (positive SE) or with a low monoculture biomass (negative SE) become dominant in species mixtures. As the CE is sensitive to variation in absolute productivity, we used the relative yield total (RYT) to investigate complementarity in relative terms (Roscher et al. 2004). RYT is the sum of the relative yields of the species in a mixture, where the relative yield is calculated as the ratio of the biomass of a species in the mixture to its biomass in monoculture (overyielding). To quantify the role of dominance, we furthermore calculated Pielou’s evenness* J* = ∑(*P*_*i*_ × ln*P*_*i*_)/ln*S*, where* P*_*i*_ is the proportional biomass of species* i* (i.e., the proportion of the mixture biomass that derives from species* i*) and* S* is the species richness of the particular mixture.

All statistical analyses were conducted using SAS 9.2 (SAS Institute Inc.). We used linear mixed models (“proc mixed”) to study the effects of plant species origin, diversity and herbivory, as well as their interactions, on aboveground, belowground, and total biomass. Block, phylogenetically adjusted species composition nested within diversity, and its interaction with origin and/or herbivory were considered as random effects. Since diversity effects (CE, SE), RYT, and evenness could only be obtained for mixtures, we applied a model with origin and herbivory as well as their interaction as fixed effects to these data. Block, phylogenetically adjusted species composition, and its interaction with origin and/or herbivory were again included as random effects. One replicate of two mixtures had to be excluded from the analyses due to misplanting of individuals, resulting in a slightly unbalanced data structure. We therefore used type 3 sum of squares for *F* tests (Shaw and Mitchell-Olds 1993). Biomass data were logarithmically transformed to approach a normal distribution and to convert multiplicative into additive effects (Rees and Brown 1992; Schädler et al. 2007).

We quantified the responses of each species to diversity and herbivory as log response ratios (LRR) of biomass to the respective treatment (Hedges et al. 1999). For this purpose, we extracted the least-square means of ln-transformed biomass values of each species from the model described above for (1) mesocosms with and without herbivory to compile the LRR to herbivory and (2) mesocosms with mixtures and monocultures to compile the LRR to diversity. The log response ratios were then calculated as LRR = ln biomass_treatment_ − ln biomass_control_. We applied a mixed model to the log response ratios with origin, functional group, and diversity (for LRR to herbivory) or herbivory (for LRR to diversity), as well as their interactions as fixed effects, and phylogenetically adjusted species composition nested within functional group as well as their interactions with origin and herbivory (or diversity) as random effects. We used the SLICE option in SAS to divide the origin × diversity and the origin × herbivory interactions into simple main effects, i.e., to test the effect of diversity or herbivory separately for native mesocosms and exotic mesocosms. It should be noted that, for a given species, the LRR to diversity is directly proportional to the logarithm of the relative yield in the mixture, which in turn contributes to CE, SE, and RYT. We preferred to analyze the LRR rather than the relative yield—which is simply a (non-logarithmized) response ratio—in order to normalize its statistical distribution, and to give deviations in the numerator the same weight as deviations in the denominator (Hedges et al. 1999).

## Results

### Productivity

**Fig. 1:**
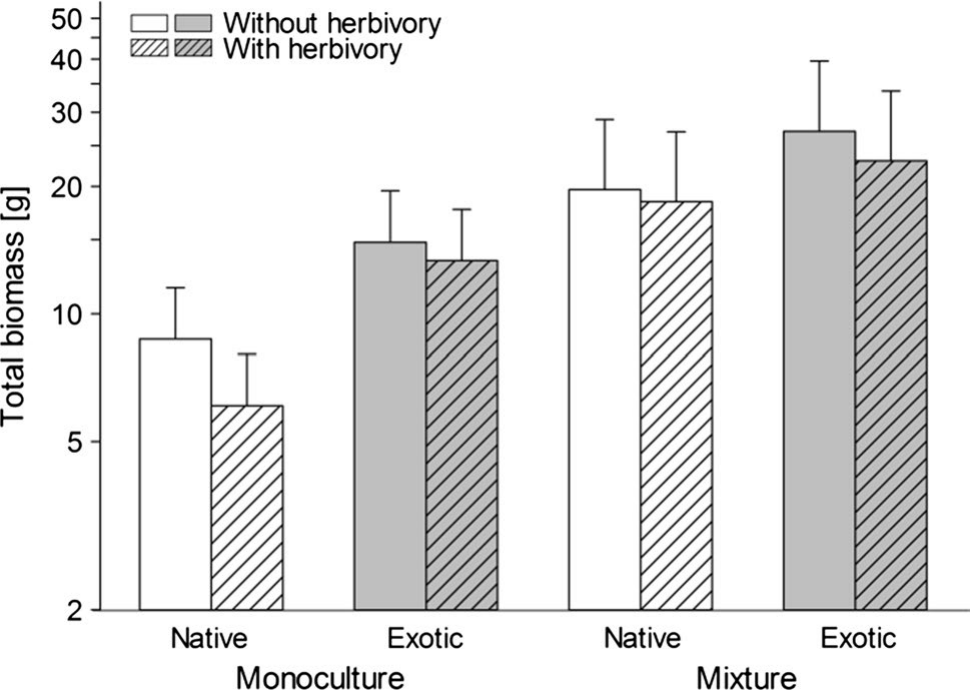
Total productivities (sum of aboveground and belowground biomass) of native and exotic species grown in monocultures and mixtures without and with herbivory. Data shown are least square means (+SE) on a logarithmic scale

Total biomass production was 48 % greater in mixtures (22.3 ± 3.2 g) than in monocultures (15.1 ± 2.5 g), indicating a positive net diversity effect (Table 2; Fig. 1). This can mainly be attributed to changes in belowground biomass (10.6 ± 1.8 vs. 7.1 ± 1.3 g), while the response of aboveground biomass was much weaker and only marginally significant (22.3 ± 3.2 vs. 15.1 ± 2.5 g). Although the diversity effects for aboveground and total biomass tended to be stronger among natives than exotics, the origin × diversity interactions were not significant. Compared to natives, exotics showed on average a 36 % higher aboveground biomass (11.4 ± 1.4 vs. 8.4 ± 1.4 g), and a 34 % higher total biomass (21.5 ± 2.4 vs. 16.0 ± 2.4 g); however, these differences were only marginally significant (Table 2). Slug grazing significantly reduced total biomass by 9 % (19.6 ± 2.7 vs. 17.8 ± 2.6 g), while its impact on aboveground biomass was only marginal, and no significant effect on belowground biomass could be detected. Monocultures and mixtures did not differ in their responses to herbivory, nor was there a difference in response between natives and exotics (no significant diversity × herbivory and origin × herbivory interactions).

**Table 2:** Results of ANOVA analyzing the effects of plant species origin (native or exotic), diversity (monoculture or mixture), herbivory (without or with), and phylogenetically adjusted species composition (nested within diversity) on aboveground, belowground, and total biomass production

Source			Biomass		
			Aboveground	Belowground	Total
Fixed effects	Num *df*	Den *df*	*F* values		
Origin (*O*)	1	16	4.43^+^	1.57	3.61^+^
Diversity (*D*)	1	16	3.90^+^	5.23*	4.55*
Herbivory (*H*)	1	16	3.54^+^	2.65	4.71*
*O* × *D*	1	16	1.02	0.14	0.63
*O* × *H*	1	16	0.76	0.02	0.30
*D* × *H*	1	16	0.51	0.22	0.56
*O* × *D* × *H*	1	16	1.95	0.16	1.39
Random effects			Variance estimates		
Phylogenetically adj. composition (*P*)			0.33^+^	0.27	0.27^+^
*O* × *P*			0.46**	0.67**	0.43**
*H* × *P*			0.02	0	0.004
*O* × *H* × *P*			0.07	0.04	0.03
Block			0.03	0.01	0.02
Residual			0.21***	0.17***	0.11***

Numerator (*Num*) and denominator (*Den*) degrees of freedom are given for fixed effects. Random effects were tested with Wald* Z* statistics. In some cases,* Z* tests could not be performed because the variance estimate was set to zero by the restricted maximum likelihood procedure

^+^ *p* < 0.10

* *p* < 0.05

** *p* < 0.01

*** *p* < 0.001

Aboveground and total biomass, but not belowground biomass, slightly varied between phylogenetically adjusted species compositions (Table 2). Random variance in all three biomass measures was to a large degree explained by variation among the phylogenetically adjusted species compositions (29 % for aboveground, 23 % for belowground, and 31 % for total biomass) and by the significant interaction of species origin with phylogenetically adjusted species composition (41 % for aboveground, 57 % for belowground, and 49 % for total biomass; Table 2). In contrast, the random interaction of phylogenetically adjusted species composition with herbivory did not help to explain random variance (no significant herbivory × phylogenetically adjusted species composition interaction, Table 2).

### Diversity effects and evenness

CE was significantly larger than zero only for the exotic mixtures without herbivory, but was not different from zero for all other mixtures (Table 3). Splitting the marginally significant interaction herbivory × species origin (Table 4) into simple main effects (“slices”) revealed a three times higher CE without herbivory than with herbivory for exotic mixtures (*F*_1,5_ = 8.03, *p* < 0.05), but no significant difference between herbivory treatments for native mixtures. In contrast to CE, the SEs of native and exotic mixtures were significantly larger (marginally significant in the case of exotics) than zero, regardless of herbivory. SE did not differ between native and exotic mixtures or between grazed and ungrazed mesocosms. Furthermore, there was no origin × herbivory interaction.

**Table 3:** Least square means (±SE) of the complementarity effect (CE), selection effect (SE), and relative yield total (RYT) for native and exotic mixtures without (H−) and with (H+) herbivory

Origin	Herbivory	CE	SE	RYT
Native	H−	0.72 ± 0.67	2.89 ± 0.86*	1.09 ± 0.07
	H+	1.13 ± 0.67	2.22 ± 0.86*	1.18 ± 0.07
Exotic	H−	2.93 ± 0.67**	1.97 ± 0.86^+^	1.30 ± 0.07***
	H+	0.75 ± 0.65	1.94 ± 0.84^+^	1.08 ± 0.06

A *t*-test was used to determine if values of the complementarity effect and selection effect differ significantly from zero, and if RYT values differ significantly from 1

^+^ *p* < 0.10

* *p* < 0.05

** *p* < 0.01

*** *p* < 0.001

**Table 4:** Results of ANOVA analyzing the effects of plant species origin (native or exotic), herbivory (without or with), and phylogenetically adjusted species composition on the complementarity effect (CE), sampling effect (SE), relative yield total (RYT), and evenness

Source			CE	SE	RYT	Evenness
Fixed effects	Num *df*	Den *df*	*F* values			
Origin (*O*)	1	5	2.83	0.54	0.59	33.38**
Herbivory (*H*)	1	5	2.64	0.45	0.77	2.34
*O* × *H*	1	5	5.67^+^	0.38	5.70^+^	0.60
Random effects			Variance estimates			
Phylogenetically adj. composition (*P*)			0	0.23	0	0
*O* × *P*			0	1.17	0	0.002
*H* × *P*			0	0	0	0
*O* × *H* × *P*			0	0	0	0
Block			0.3	0.43	0	0.002
Residual			3.31***	3.04***	0.04***	0.007***

Numerator (*Num*) and denominator (*Den*) degrees of freedom are given for fixed effects. Random effects were tested with Wald *Z* statistics. In some cases *Z* tests could not be performed because the variance estimate was set to zero by the restricted maximum likelihood procedure

^+^ *p* < 0.10

* *p* < 0.05

** *p* < 0.01

*** *p* < 0.001

**Fig. 2:**
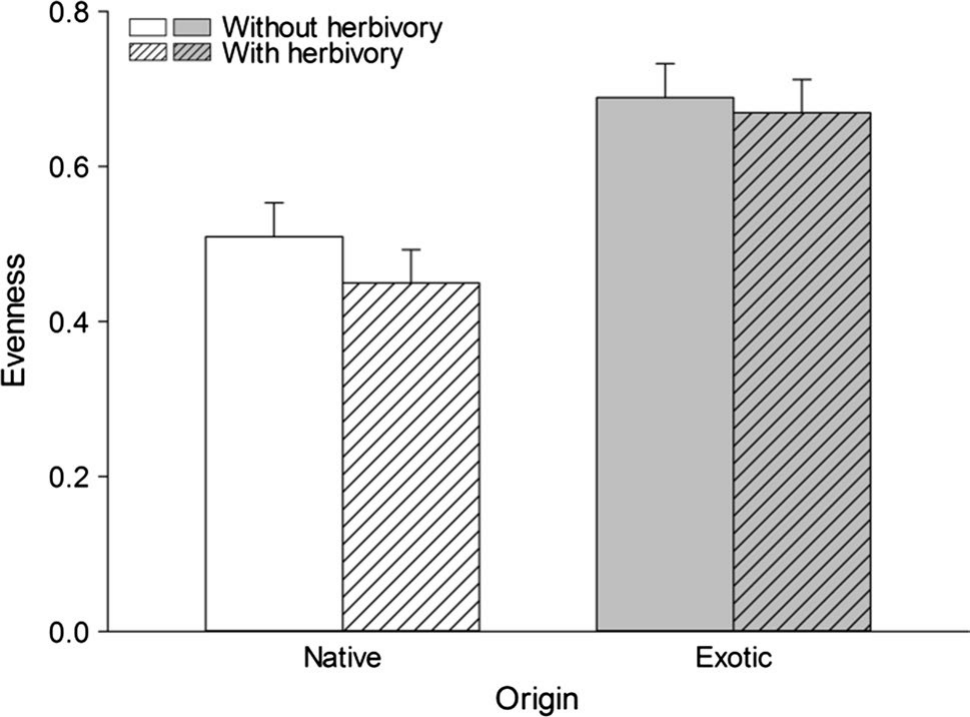
Evenness values of native and exotic mixtures without and with herbivory. Data shown are least-square means (+SE)

Similar to CE, RYT was significantly larger for the exotic than the native mesoscosms only in the absence of herbivory (Table 3), resulting in a marginally significant origin × herbivory interaction (Table 4). Hence, the large CEs of these mixtures were caused by an average increase in biomass of the component species relative to their monocultures, rather than by their high productivity per se. Exotic mixtures showed a much higher evenness than native mixtures (Fig. 2), while herbivory had no effect on evenness (Table 4). Phylogenetically adjusted species composition did not contribute to the variance in CE and SE or evenness, as most of the random variation was attributed to residual variance (Table 4).

### Responses of species and functional groups

**Fig. 3:**
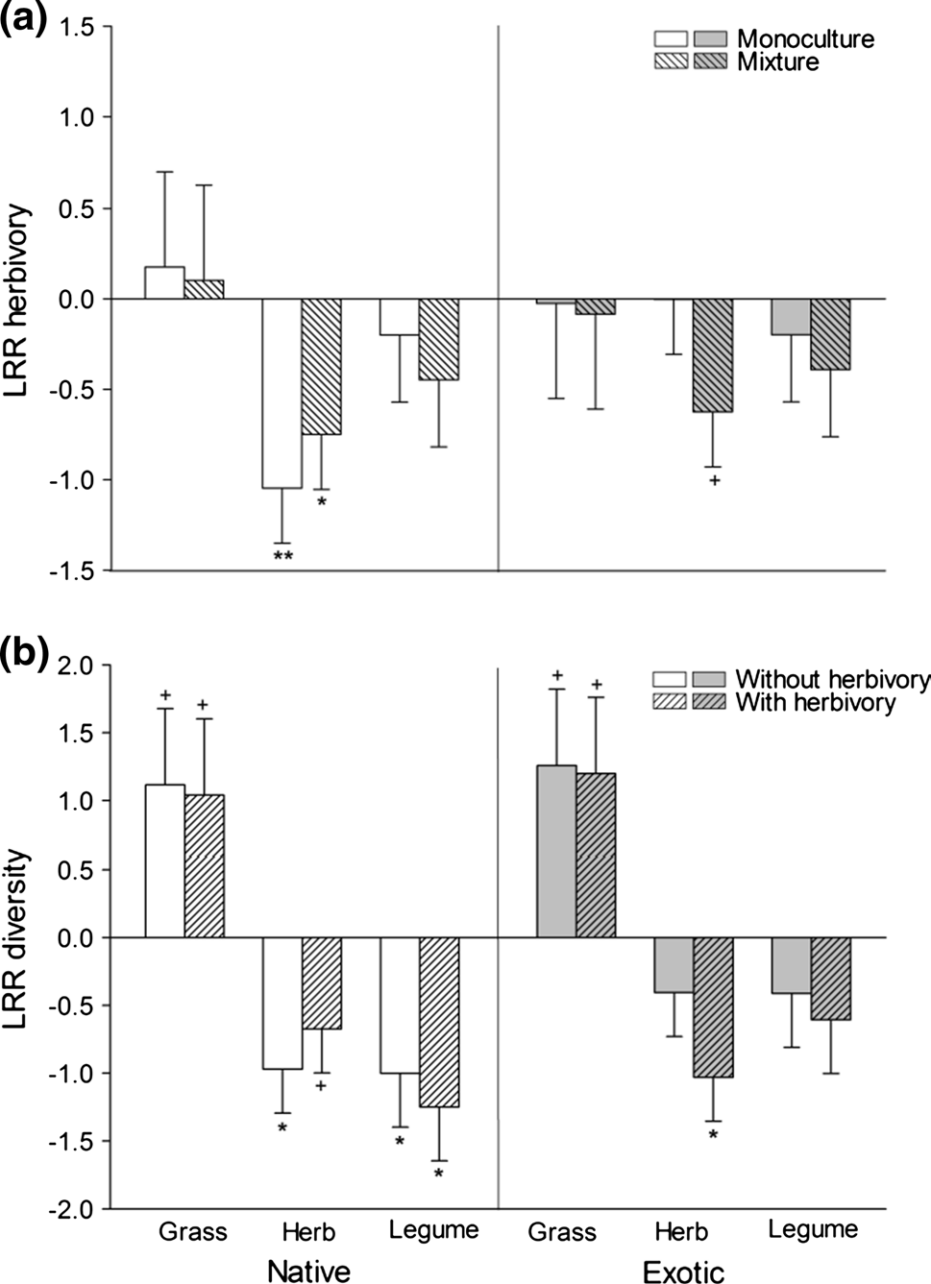
Log response ratios (LRR) of biomass for native and exotic grasses, nonleguminous herbs, and legumes **a** grown in monoculture and mixture in response to herbivory and **b** grown without and with herbivory in response to diversity. *Positive* *values* indicate an increase in performance in the herbivory treatment and in the mixture, respectively. *Symbols* *on* *bars* indicate the significance of responses; + *p* < 0.10, * *p* < 0.05

The analysis of LRR across all 24 species revealed that the three functional groups—grasses, legumes, and nonleguminous herbs—responded differently to diversity (Fig. 3, Online resource 2 in the ESM). Grasses showed on average the strongest positive response to diversity (LRR 1.15 ± 0.48), while leguminous herbs and nonleguminous herbs responded negatively (−0.77 ± 0.28 and −0.82 ± 0.34). There was a marginally significant origin × functional group × herbivory interaction: without herbivory and consistent with the high CE and RYT values in this treatment, exotic grasses showed a large positive LRR to diversity, while the (slightly negative) responses of exotic nonleguminous herbs and legumes did not deviate from zero. With herbivory, exotic grasses responded positively as well, but this effect was offset by a large negative response of nonleguminous herbs to diversity. Similar to exotic grasses, native grasses responded positively to diversity, while native herbs and legumes responded negatively both with and without herbivory. The contribution of phylogenetically adjusted species composition to the random variation in LRR to diversity was marginal (Online resource 2 in the ESM).

Neither species origin nor functional group had an effect on LRR to herbivory, but there was a marginally significant origin × functional group × diversity interaction (Online resource 2 in the ESM). The slightly negative responses of exotic grasses and legumes did not differ from zero in monocultures and mixtures, while exotic nonleguminous herbs showed a stronger negative response to slug grazing in mixtures but not in monocultures. In contrast, native nonleguminous herbs showed a large negative response to herbivory both in monocultures and mixtures, while the slightly negative response of native grasses and legumes was again not different from zero. The contribution of phylogenetically adjusted species composition to the random variation in LRR to herbivory was marginal (Online resource 2 in the ESM). Using ANCOVA, we checked if the responses of individual species to slug herbivory were dependent on their proportional contributions to the biomass of the mixture (as measured in the without-herbivory treatment). Species with high proportional biomasses in mixtures (e.g., the grasses) were indeed found to be least affected by slug grazing, while species with low proportional biomasses were strongly suppressed (*r*^2^ = 0.23, *p* < 0.05). Native and exotic species did not differ with respect to the relationship between proportional biomass and LRR to herbivory (*F*_1, 20_ = 0.06, *p* = 0.81).

## Discussion

The positive relationship between diversity and productivity encountered in our experiments confirms previous results from other biodiversity experiments (Hector et al. 1999; Hooper et al. 2005; Cardinale et al. 2011). The results of this short-term experimental mesocosm study suggest that higher diversity increases productivity, as also seen in long-term field studies.

Moreover, in our experiment, the processes underlying the positive effect of diversity on productivity differed between exotic and native communities, but not in the way we expected them to do. Evidence is increasing that plant communities are not only the result of assembly processes acting on species with pre-defined niches, but that plant–plant interactions can also cause evolutionary processes leading to niche partitioning or facilitation among coexisting species (Thorpe et al. 2011). This suggests that the complementarity effect (CE) should be higher among native species that share a co-evolutionary history, while the selection effect (SE) should be stronger among exotic species that are not likely to share a co-evolutionary history (Wilsey et al. 2009). However, the positive net diversity effect in our study resulted from exactly the opposite pattern, i.e., a stronger SE in native mixtures and a stronger CE in exotic mixtures. The higher CE in exotic mixtures was also reflected in a higher evenness compared with native counterparts.

The much stronger SE in observed native communities suggests that interspecific competition among native species may be even larger than that among exotics, at least in the early stages of community development, thereby resulting in lower evenness. However, the higher importance of SE versus CE for native mixtures in our experiment coincides with results from other biodiversity experiments, which highlight the greater importance of SE in the early stages and of CE in the later stages of community development (Cardinale et al. 2007; Fargione et al. 2007; Marquard et al. 2009; Reich et al. 2012). It is, however, far from clear whether this pattern holds true for exotic species, since very few experiments testing for diversity effects in exotic communities have been reported (but see Wilsey et al. 2009; Isbell and Wilsey 2011; Cook-Patton and Agrawal 2014). Wilsey et al. (2009) revealed, after an experimental period of 2 years, that exotic communities decreased dramatically in diversity, which was attributed to a much higher SE compared to native communities. In accordance with the study by Cook-Patton and Agrawal (2014), we found a higher CE in exotic than in native mixtures, but, in contrast to their results, we encountered a significant SE in both native and exotic mixtures.

In our experiment, the high CE accompanied by a high evenness in exotic communities may also suggest that these novel ecosystems are not necessarily dominated by greater interspecific competition in early community stages. There are several possible explanations for the high CE, which is simultaneously influenced by various interactions among species (Loreau et al. 2012) such as niche partitioning and relative fitness differences (Carroll et al. 2011). First, some of our exotic species originate from the same region (out of 12 species, 7 are native to the Mediterranean region and 8 originate from western Asia; Table 1) and may therefore share a co-evolutionary history. Although we do not have information about their spatial co-occurrence, it is possible that these species may have undergone a niche partitioning process which they are able to benefit from in non-native regions (Thorpe et al. 2011). Second, CE is reduced by any variability in relative fitness among species (Carroll et al. 2011). As our native species showed a much larger variation in biomass than our exotic species (coefficient of variation among species: 172 % within native mixtures vs. 121 % within exotic mixtures), this may have contributed to the higher CE of exotics as well. Third, another explanation might be that the CE observed in our study may be confined to early stages of community development. Such transient CE can occur in unstable species mixtures when niche differences among species are present but are not strong enough to overcome fitness differences (Turnbull et al. 2013). We therefore suggest that accompanying long-term studies using the same species pool would help to disentangle transient from long-term effects. Other reasons may involve a more efficient uptake and/or use of soil nutrients by exotics compared to native species (Mack et al. 2001).

Our finding of a higher productivity in exotic communities is consistent with the results of the few other reported studies comparing the productivities of native and exotic-dominated plant communities (Maron and Marler 2008; Wilsey et al. 2009; Maron et al. 2014). This species origin effect was not, however, as strong as in previously reported field experiments, perhaps due to the short duration of our study, and would presumably be more pronounced in a long-term experiment. The higher biomass of exotics (in monoculture as well as in a mixture) compared to natives also agrees with the findings of a recent meta-analysis: that invasive exotics attain significantly larger individual plant sizes than natives (Van Kleunen et al. 2010).

Competitive advantages of exotic species over native species are often attributed to the release of these species, particularly from specialist herbivores (Keane and Crawley 2002). However, the enemy release hypothesis also proposes a reduced attack on exotics by generalist herbivores in the invaded range, but experimental evidence for this part of the hypothesis is rare (but see Peters 2007; Motheral and Orrock 2010). In our study, we found no evidence for reduced grazing by slugs on exotic plant species or exotic mixtures compared to native ones. Furthermore, the higher productivity of exotic species compared to native species was not related to differential feeding of *Arion vulgaris*, and slug grazing had no effect on the evenness of exotic and native communities. Our results showing equal responses of exotic and native grassland species to slug herbivory are consistent with field studies of the effects of generalist herbivores on the establishment of exotic vs. native seedlings (Strauss et al. 2009) or leaf damage (Agrawal and Kotanen 2003).

However, in the absence of slug grazing, exotic but not native mixtures showed a threefold higher CE. The process behind this effect becomes evident when we inspect the responses of the three functional groups: both with and without herbivory, native legumes and nonleguminous herbs showed a strong negative response to mixing with other species, i.e., they suffered more from interspecific than from intraspecific competition. In contrast, exotic legumes and nonleguminous herbs were much less affected by interspecific competition, but only when herbivores were absent. In the presence of herbivores, however, the biomass of exotic nonleguminous herbs was strongly reduced by interspecific competition. Accordingly, the impact of slug herbivory on exotic nonleguminous herbs was stronger in mixtures than in monocultures. We can only speculate about the reasons for the differential responses of exotic versus native herbs and legumes. Since the exotic legumes used for our experiment were introduced to Central Europe as fodder crops (Table 1; Hanelt 2001; Kühn et al. 2004), they should be less resistant to, and hence preferred by, generalist herbivores (Isbell and Wilsey 2011). Yet, owing to a trade-off between resistance and tolerance (Strauss and Agrawal 1999; Leimu and Koricheva 2006), they may show a pronounced compensatory ability, which could explain their weak net response to herbivory. Exotic herbs—subordinate species in our mixtures—may in turn suffer more from herbivory because of their smaller stature on average. It should be noted that the observed response of plant species to herbivory we measured in our experiment is the net effect of both resistance and tolerance to herbivory. Resistance, defined as “any plant trait that reduces the preference or performance of herbivores,” and tolerance, defined as “the degree to which plant fitness is affected by herbivore damage relative to fitness in the undamaged state” (Strauss and Agrawal 1999), cannot be separated in our experiment.

Independent of their origin, grasses suffered least from slug grazing. Subordinate species, i.e., most of the nonleguminous herbs, which showed rather negative effects on interspecific competition in mixtures, also responded negatively to herbivory, while species with a positive response to interspecific competition, i.e., all grasses, responded positively to herbivory. Obviously, the high competitive ability of grasses was amplified by their resistance to generalist herbivores, e.g., owing to their high silica concentration, and/or their compensatory ability, leading to a competitive advantage over neighboring species (Hanley et al. 1996; Wilby and Brown 2001).

To conclude, although we cannot extrapolate our results to later stages of community development, for which long-term field experiments would be more appropriate, our study represents one of the very few reported studies to experimentally compare the diversity–productivity relationship and the impact of herbivory on this relationship between exotic and native communities. While the studies of Wilsey et al. (2009, 2011) and Cook-Patton and Agrawal (2014) and our study consistently found a higher productivity of exotic than native mixtures (but see Isbell and Wilsey 2011), they suggested opposing effects of CE and SE on the diversity–productivity relationship in these communities. We can only speculate about the reasons for this discrepancy beyond the possibility that our study might simply reflect transient effects. One reason could be peculiarities of the species pools used for the experiments: in our study, for instance, variation in biomass was much lower among exotic species than among native species, which may in turn influence the magnitude of CE and SE. Moreover, the larger CE of exotic mixtures, which was only observed in the absence of herbivores in our experiment, cannot be compared to the results of Wilsey et al. (2009, 2011) and Cook-Patton and Agrawal (2014), as there was no herbivore exclusion treatment in their studies. Independent of these discrepancies, the differences in diversity effects between native and exotic communities suggest that researchers should be cautious when deriving conclusions from experiments considering one species origin only.

## Electronic supplementary material

Below is the link to the electronic supplementary material.

Supplementary material 1 (PDF 179 kb)

## References

[CR1] Agrawal AA, Kotanen PM (2003). Herbivores and the success of exotic plants: a phylogenetically controlled experiment. Ecol Lett.

[CR2] Allan E, Crawley MJ (2011). Contrasting effects of insect and molluscan herbivores on plant diversity in a long-term field experiment. Ecol Lett.

[CR3] Altermann M, Rinklebe J, Merbach I, Körschens M, Langer U, Hofmann B (2005) Chernozem—soil of the year 2005. J Plant Nutr Soil Sci 168:725–740

[CR4] Bell T, Lilley AK, Hector A, Schmid B, King L, Newman JA (2009) A linear model method for biodiversity–ecosystem functioning experiments. Am Nat 174:836–849 10.1086/647931 19842969

[CR5] Bruelheide H, Scheidel U (1999). Slug herbivory as a limiting factor for the geographical range of *Arnica montana*. J Ecol.

[CR6] Buschmann H, Keller M, Porret N, Dietz H, Edwards PJ (2005). The effect of slug grazing on vegetation development and plant species diversity in an experimental grassland. Funct Ecol.

[CR7] Cardinale BJ, Wright JP, Cadotte MW, Carroll IT, Hector A, Srivastava DS, Loreau M, Weis JJ (2007) Impacts of plant diversity on biomass production increase through time because of species complementarity. Proc Natl Acad Sci USA 104:18123–18128 10.1073/pnas.0709069104 PMC2084307 17991772

[CR8] Cardinale BJ, Matulich KL, Hooper DU, Byrnes JE, Duffy E, Gamfeldt L, Balvanera P, O’Connor MI, Gonzalez A (2011). The functional role of producer diversity in ecosystems. Am J Bot.

[CR9] Carroll IT, Cardinale BJ, Nisbet RM (2011). Niche and fitness differences relate the maintenance of diversity to ecosystem function. Ecology.

[CR10] Carson WP, Root RB (2000). Herbivory and plant species coexistence: community regulation by an outbreaking phytophagous insect. Ecol Monogr.

[CR11] Cook-Patton SC, Agrawal AA (2014). Exotic plants contribute positively to biodiversity functions but reduce native seed production and arthropod richness. Ecology.

[CR12] Cook-Patton SC, LaForgia M, Parker JD (2014) Positive interactions between herbivores and plant diversity shape forest regeneration. Proc R Soc Lond Ser B Biol Sci 281:20140261 10.1098/rspb.2014.0261 PMC3996617 24718763

[CR13] Duffy JE, Cardinale BJ, France KE, McIntyre PB, Thébault E, Loreau M (2007). The functional role of biodiversity in ecosystems: incorporating trophic complexity. Ecol Lett.

[CR14] Dukes JS (2001). Productivity and complementarity in grassland microcosms of varying diversity. Oikos.

[CR15] Fargione J, Tilman D, Dybzinski R, Lambers JHR, Clark C, Harpole WS, Knops JM, Reich PB, Loreau M (2007). From selection to complementarity: shifts in the causes of biodiversity–productivity relationships in a long-term biodiversity experiment. Proc R Soc Lond Ser B Biol Sci.

[CR16] Fridley JD (2002). Resource availability dominates and alters the relationship between species diversity and ecosystem productivity in experimental plant communities. Oecologia.

[CR17] Hallett SG (2006). Dislocation from coevolved relationships: a unifying theory for plant invasion and naturalization?. Weed Sci.

[CR18] Hanelt P (2001). Mansfeld’s encyclopedia of agricultural and horticultural crops.

[CR19] Hanley M (2004). Seedling herbivory and the influence of plant species richness in seedling neighbourhoods. Plant Ecol.

[CR20] Hanley M, Fenner M, Edwards P (1996). Mollusc grazing and seedling survivorship of four common grassland plant species: the role of gap size, species and season. Acta Oecol.

[CR21] Hector A, Schmid B, Beierkuhnlein C, Caldeira M, Diemer M, Dimitrakopoulos P, Finn J, Freitas H, Giller P, Good J (1999). Plant diversity and productivity experiments in European grasslands. Science.

[CR22] Hedges LV, Gurevitch J, Curtis PS (1999). The meta-analysis of response ratios in experimental ecology. Ecology.

[CR23] Hejda M, Pyšek P, Jarošík V (2009). Impact of invasive plants on the species richness, diversity and composition of invaded communities. J Ecol.

[CR24] Hillebrand H, Gruner DS, Borer ET, Bracken ME, Cleland EE, Elser JJ, Harpole WS, Ngai JT, Seabloom EW, Shurin JB (2007) Consumer versus resource control of producer diversity depends on ecosystem type and producer community structure. Proc Natl Acad Sci USA 104:10904–10909 10.1073/pnas.0701918104 PMC1904146 17581875

[CR25] Hooper D, Chapin Iii F, Ewel J, Hector A, Inchausti P, Lavorel S, Lawton J, Lodge D, Loreau M, Naeem S (2005). Effects of biodiversity on ecosystem functioning: a consensus of current knowledge. Ecol Monogr.

[CR26] Hulme PE (1994) Seedling herbivory in grassland: relative impact of vertebrate and invertebrate herbivores. J Ecol 82:873–880

[CR27] Isbell FI, Wilsey BJ (2011). Increasing native, but not exotic, biodiversity increases aboveground productivity in ungrazed and intensely grazed grasslands. Oecologia.

[CR28] Joshi J, Vrieling K (2005). The enemy release and EICA hypothesis revisited: incorporating the fundamental difference between specialist and generalist herbivores. Ecol Lett.

[CR29] Keane RM, Crawley MJ (2002). Exotic plant invasions and the enemy release hypothesis. Trends Ecol Evol.

[CR30] Keller M, Kollmann J, Edwards PJ (1999). Palatability of weeds from different European origins to the slugs *Deroceras reticulatum* Müller and *Arion lusitanicus* Mabille. Acta Oecol.

[CR31] Koricheva J, Vehviläinen H, Riihimäki J, Ruohomäki K, Kaitaniemi P, Ranta H (2006). Diversification of tree stands as a means to manage pests and diseases in boreal forests: myth or reality?. Can J For Res.

[CR32] Kühn I, Durka W, Klotz S (2004). BiolFlor—a new plant-trait database as a tool for plant invasion ecology. Divers Distrib.

[CR33] Leimu R, Koricheva J (2006). A meta-analysis of tradeoffs between plant tolerance and resistance to herbivores: combining the evidence from ecological and agricultural studies. Oikos.

[CR34] Liao Z-Y, Zheng Y-L, Lei Y-B, Feng Y-L (2014). Evolutionary increases in defense during a biological invasion. Oecologia.

[CR35] Loreau M, Hector A (2001). Partitioning selection and complementarity in biodiversity experiments. Nature.

[CR36] Loreau M, Sapijanskas J, Isbell F, Hector A (2012). Niche and fitness differences relate the maintenance of diversity to ecosystem function: comment. Ecology.

[CR37] Mack MC, D’Antonio CM, Ley RE (2001). Alteration of ecosystem nitrogen dynamics by exotic plants: a case study of C4 grasses in Hawaii. Ecol Appl.

[CR38] Maron JL, Marler M (2008). Effects of native species diversity and resource additions on invader impact. Am Nat.

[CR39] Maron JL, Marler M, Klironomos JN, Cleveland CC (2011). Soil fungal pathogens and the relationship between plant diversity and productivity. Ecol Lett.

[CR40] Maron JL, Auge H, Pearson DE, Korell L, Hensen I, Suding KN, Stein C (2014). Staged invasions across disparate grasslands: effects of seed provenance, consumers and disturbance on productivity and species richness. Ecol Lett.

[CR41] Marquard E, Weigelt A, Temperton VM, Roscher C, Schumacher J, Buchmann N, Fischer M, Weisser WW, Schmid B (2009). Plant species richness and functional composition drive overyielding in a six-year grassland experiment. Ecology.

[CR42] Motheral SM, Orrock JL (2010). Gastropod herbivore preference for seedlings of two native and two exotic grass species. Am Midl Nat.

[CR43] Mulder Koricheva, Huss D, Högberg Joshi (1999). Insects affect relationships between plant species richness and ecosystem processes. Ecol Lett.

[CR44] Olden JD, LeRoy Poff N, Douglas MR, Douglas ME, Fausch KD (2004). Ecological and evolutionary consequences of biotic homogenization. Trends Ecol Evol.

[CR45] Olff H, Ritchie ME (1998). Effects of herbivores on grassland plant diversity. Trends Ecol Evol.

[CR46] Ortega YK, Pearson DE (2005). Weak vs. strong invaders of natural plant communities: assessing invasibility and impact. Ecol Appl.

[CR47] Peters HA (2007). The significance of small herbivores in structuring annual grassland. J Veg Sci.

[CR48] Pfenninger M, Weigand A, Bálint M, Klussmann-Kolb A (2014). Misperceived invasion: the Lusitanian slug (*Arion lusitanicus* auct. non-Mabille or *Arion vulgaris* Moquin-Tandon 1855) is native to Central Europe. Evol Appl.

[CR49] Rees M, Brown VK (1992) Interactions between invertebrate herbivores and plant competition. J Ecol 80:353–360

[CR50] Reich PB, Knops J, Tilman D, Craine J, Ellsworth D, Tjoelker M, Lee T, Wedin D, Naeem S, Bahauddin D (2001). Plant diversity enhances ecosystem responses to elevated CO_2_ and nitrogen deposition. Nature.

[CR51] Reich PB, Tilman D, Isbell F, Mueller K, Hobbie SE, Flynn DF, Eisenhauer N (2012). Impacts of biodiversity loss escalate through time as redundancy fades. Science.

[CR52] Roscher C, Schumacher J, Baade J, Wilcke W, Gleixner G, Weisser WW, Schmid B, Schulze E-D (2004). The role of biodiversity for element cycling and trophic interactions: an experimental approach in a grassland community. Basic Appl Ecol.

[CR53] Sax DF, Gaines SD (2003). Species diversity: from global decreases to local increases. Trends Ecol Evol.

[CR54] Schädler M, Brandl R, Haase J (2007). Antagonistic interactions between plant competition and insect herbivory. Ecology.

[CR55] Scheidel U, Bruelheide H (1999). Selective slug grazing on montane meadow plants. J Ecol.

[CR56] Scherber C, Mwangi PN, Temperton VM, Roscher C, Schumacher J, Schmid B, Weisser WW (2006). Effects of plant diversity on invertebrate herbivory in experimental grassland. Oecologia.

[CR57] Schnitzer SA, Klironomos JN, HilleRisLambers J, Kinkel LL, Reich PB, Xiao K, Rillig MC, Sikes BA, Callaway RM, Mangan SA (2011). Soil microbes drive the classic plant diversity–productivity pattern. Ecology.

[CR58] Schuldt A, Baruffol M, Böhnke M, Bruelheide H, Härdtle W, Lang AC, Nadrowski K, Von Oheimb G, Voigt W, Zhou H (2010). Tree diversity promotes insect herbivory in subtropical forests of south-east China. J Ecol.

[CR59] Shaw RG, Mitchell-Olds T (1993) ANOVA for unbalanced data: an overview. Ecology 74:1638–1645

[CR60] Stein C, Auge H, Fischer M, Weisser WW, Prati D (2008). Dispersal and seed limitation affect diversity and productivity of montane grasslands. Oikos.

[CR61] Stein C, Unsicker SB, Kahmen A, Wagner M, Audorff V, Auge H, Prati D, Weisser WW (2010). Impact of invertebrate herbivory in grasslands depends on plant species diversity. Ecology.

[CR62] Strauss SY, Agrawal AA (1999). The ecology and evolution of plant tolerance to herbivory. Trends Ecol Evol.

[CR63] Strauss SY, Stanton ML, Emery NC, Bradley CA, Carleton A, Dittrich-Reed DR, Ervin OA, Gray LN, Hamilton AM, Rogge JH (2009). Cryptic seedling herbivory by nocturnal introduced generalists impacts survival, performance of native and exotic plants. Ecology.

[CR64] Thébault E, Loreau M (2005). Trophic interactions and the relationship between species diversity and ecosystem stability. Am Nat.

[CR65] Thorpe AS, Aschehoug ET, Atwater DZ, Callaway RM (2011). Interactions among plants and evolution. J Ecol.

[CR66] Turnbull LA, Levine JM, Loreau M, Hector A (2013). Coexistence, niches and biodiversity effects on ecosystem functioning. Ecol Lett.

[CR67] Van Kleunen M, Weber E, Fischer M (2010). A meta-analysis of trait differences between invasive and non-invasive plant species. Ecol Lett.

[CR68] Vilà M, Espinar JL, Hejda M, Hulme PE, Jarošík V, Maron JL, Pergl J, Schaffner U, Sun Y, Pyšek P (2011). Ecological impacts of invasive alien plants: a meta-analysis of their effects on species, communities and ecosystems. Ecol Lett.

[CR69] Wilby A, Brown V (2001). Herbivory, litter and soil disturbance as determinants of vegetation dynamics during early old-field succession under set-aside. Oecologia.

[CR70] Wilsey BJ, Teaschner TB, Daneshgar PP, Isbell FI, Polley HW (2009). Biodiversity maintenance mechanisms differ between native and novel exotic-dominated communities. Ecol Lett.

[CR71] Wilsey BJ, Daneshgar PP, Polley HW (2011). Biodiversity, phenology and temporal niche differences between native and novel exotic-dominated grasslands. Perspect Plant Ecol Evol Syst.

[CR72] Wilsey BJ, Daneshgar PP, Hofmockel K, Polley HW (2014). Invaded grassland communities have altered stability-maintenance mechanisms but equal stability compared to native communities. Ecol Lett.

