# Supplementary material for: Mechanisms driving diversity–productivity relationships differ between exotic and native communities and are affected by gastropod herbivory
Source: Oecologia. 2015 Aug 4;180:1025–36. doi: 10.1007/s00442-015-3395-2 (PMC4819496; doi:10.1007/s00442-015-3395-2)
Supplement: Supplementary file 1 — Supplementary material 1 (PDF 179 kb) [file 442_2015_3395_MOESM1_ESM.pdf]

ELECTRONIC SUPPLEMENTARY MATERIAL (ESM)

Mechanisms driving diversity-productivity relationships differ between exotic and native communities, and are affected by gastropod herbivory

Lotte Korell*, Robin Schmidt, Helge Bruelheide, Isabell Hensen, Harald Auge

***Corresponding author;** E-mail: lo.korell@gmail.com

Online resource 1. Phylogenetically adjusted species compositions of the exotic and native mixtures.

Origin	Functional group	Species	Native mixtures						Exotic mixtures					
			1	2	3	4	5	6	1	2	3	4	5	6
Native	Grasses	*Bromus hordeaceus*	x			x	x							
		Dactylis glomerata		x	x			x						
	Legumes	*Medicago falcata*	x		x		x							
		Onobrychis arenaria	x			x		x						
		Vicia cracca		x	x			x						
		Lotus corniculatus		x		x	x							
	Non-legume herbs	*Falcaria vulgaris*		x		x	x							
		Pimpinella saxifraga	x			x		x						
		Inula salicina	x		x		x							
		Tragopogon dubius		x	x			x						
		Sanguisorba minor ssp. *minor*		x	x		x							
		Dianthus carthusianorum	x			x		x						
Exotic	Grasses	*Bromus tectorum*							x			x	x	
		Lolium multiflorum								x	x			x
	Legumes	*Medicago x varia*							x		x		x	
		Onobrychis viciifolia							x			x		x
		Vicia villosa								x	x			x
		Lupinus polyphyllus								x		x	x	
	Non-legume herbs	*Foeniculum vulgare*								x		x	x	
		Pimpinella peregrina							x			x		x
		Senecio inaequidens							x		x		x	
		Solidago canadensis								x	x			x
		Sanguisorba minor ssp. *polygama*								x	x		x	
		Dianthus giganteus							x			x		x

Online resource 2. Results of ANOVA analyzing a) the effects of origin, herbivory, functional group and phylogenetically adjusted species composition (nested within functional group) on the log response ratio (LRR) to diversity, b) the effect of origin, diversity, functional group and phylogenetically adjusted species composition (nested within functional group) on the log response ratio (LRR) to herbivory. Numerator (Num) and denominator (Den) degrees of freedom are given for fixed effects. Random effects were tested with a Wald Z statistics. Significance levels are $^{+}$ P<0.10, * P<0.05, ** P<0.01, *** P<0.001

a)

Source			LRR diversity
Fixed effects	Num d.f.	Den d.f.	F-values
Origin [O]	1	9	2.34
Herbivory [H]	1	9	1.26
Functional group [FG]	1	9	6.66*
O × H	2	9	1.93
FG × O	2	9	0.95
FG × H	2	9	0.09
FG × O × H	2	9	3.39^{+}
Random effects			Variance estimates
Phylogenetically adj. composition [P]			0.36^{+}
O × P			0.12^{+}
H × P			0.03
Residual			0.10***

b)

Source			LRR herbivory
Fixed effects	Num d.f.	Den d.f.	F-values
Origin [O]	1	9	0.36
Diversity [D]	1	9	1.26
Functional group [FG]	1	9	0.97
O × D	2	9	1.93
FG × O	2	9	0.97
FG × D	2	9	0.09
FG × O × D	2	9	3.39^{+}
Random effects			Variance estimates
Phylogenetically adj. composition [P]			0.20
O × P			0.21*
D × P			0.03
Residual			0.10***
